# Vigorous physical activity in relation to family affluence: time trends in Europe and North America

**DOI:** 10.1007/s00038-019-01271-8

**Published:** 2019-07-05

**Authors:** Dagmar Sigmundová, Erik Sigmund, Riki Tesler, Kwok W. Ng, Zdenek Hamrik, Frida Kathrine Sofie Mathisen, Jo Inchley, Jens Bucksch

**Affiliations:** 1 Institute of Active Lifestyle, Faculty of Physical Culture, Palacký University Olomouc, Tr. Miru 117, 77111 Olomouc, Czech Republic; 2 Department of Health Systems Management, Faculty of Health Sciences, Ariel University, Ariel, Israel; 3 Department of Physical Education and Sport Sciences, University of Limerick, Limerick, Ireland; 4 School of Educational Sciences and Psychology, University of Eastern Finland, Joensuu, Finland; 5 Department of Recreation and Leisure Studies, Faculty of Physical Culture, Palacký University Olomouc, Olomouc, Czech Republic; 6 Department of Health Promotion and Development, Faculty of Psychology, University of Bergen, Bergen, Norway; 7 MRC/CSO Social and Public Health Sciences Unit, University of Glasgow, Glasgow, UK; 8 School of Medicine, University of St. Andrews, North Haugh, St. Andrews, UK; 9 Department of Prevention and Health Promotion, Faculty of Natural and Human Sciences, Heidelberg University of Education, Heidelberg, Germany

**Keywords:** Vigorous physical activity, HBSC study, Trends, Family affluence

## Abstract

**Objectives:**

The aim of the study was to determine secular trends in vigorous physical activity (VPA) among adolescents in relation to family affluence across 34 countries.

**Methods:**

This study used data from the Health Behaviour in School-aged Children (HBSC) study from 34 countries in Europe and North America. Adolescents (*N* = 501,647) aged 11, 13 and 15 years across three survey cycles (2006, 2010, 2014) self-reported data on VPA and a family affluence scale (FAS) using standardized questionnaires.

**Results:**

A significant increase in VPA was found in low-FAS boys (girls) in four (10) countries and a decrease in four (three) countries. In high-FAS boys (girls), a significant increase was observed in nine (11) countries and a decrease in two(three) countries. An overall significant increase in meeting the VPA recommendations was found in high-FAS boys (OR 1.11; 95% CI 1.06–1.16) and in all FAS groups in girls, with the largest effect being found among high-FAS girls (OR 1.24; 95% CI 1.18–1.30).

**Conclusions:**

A country-specific increase in VPA was observed primarily in the medium- and high-FAS categories. This study suggests a need to focus on increasing VPA efforts, especially in low- and medium-FAS boys.

## Introduction

Despite the well-established benefits of physical activity (PA), a substantial proportion of children and adolescents is not physically active enough to improve their health (Hallal et al. 2012). PA levels decline from childhood to adolescence, especially among girls (Cairney et al. 2014; Wichstrom et al. 2013). Understanding recent trends in adolescent PA is important for the promotion of health-related PA and to ensure that appropriate interventions can be put in place to support adolescent health and well-being (Ekelund et al. 2016). PA includes a wide spectrum of activities of different intensity, ranging from light PA and moderate-to-vigorous PA (MVPA) to vigorous PA (VPA), with varying impacts on human health (Ekelund et al. 2012; Chaput et al. 2013). Evidence suggests that the greatest health benefits are associated with VPA (Hay et al. 2012; Parikh and Stratton 2011; Steele et al. 2009). VPA correlates significantly with children’s and adolescents’ fitness (Parikh and Stratton 2011), and is a significant predictor of their fatness (Hay et al. 2012; Parikh and Stratton 2011; Steele et al. 2009). Moreover, compared with lower-intensity activity, VPA in adolescents is also more strongly associated with self-esteem, self-efficacy and other positive psychological and social health outcomes (Eime et al. 2013).

Few studies have examined trends in VPA and these are usually based on data collected before 2010 (Irving et al. 2003; Samdal et al. 2007). The results from previous studies do not indicate a clear trend in VPA across countries or the age or gender of adolescents (Irving et al. 2003; Samdal et al. 2007). The latest pan-European VPA trend-related study, involving over 35 countries, identifies a clearer increasing trend in participation in VPA among girls than boys (Inchley et al. 2017). Although it has been documented that family affluence is related to VPA, with adolescents from more affluent families being more likely to participate in VPA in many countries and regions, a more detailed analysis of family affluence and VPA is still lacking (Inchley et al. 2017) particularly from a cross-national perspective.

Thus, our aim was to analyse current international trends in VPA and to examine whether trends differ according to family affluence across 34 countries between 2006 and 2014. The findings will provide a better understanding of changes in adolescent VPA over time and highlight where social inequalities in VPA exist to inform future programmes and policies with the aim of increasing time spent on VPA and to identify target groups with the highest need for the promotion of VPA.

## Methods

### Study design and participants

This study used data from 34 countries participating in the World Health Organization collaborative cross-national Health Behaviour in School-aged Children (HBSC) study in the 2006, 2010 and 2014 survey cycles. HBSC is an international study with a standardized methodological approach (Currie et al. 2011, 2014). Schools were the primary sampling unit and were selected randomly. Participation in the study was anonymous and voluntary. Active or passive consent was sought from school administrators, parents and children, as per national human participant requirements. All the survey procedures for each data collection cycle were documented and can be downloaded from http://www.hbsc.org/methods/index.html. Ethical approvals for the study were obtained at a national or regional level; each country obtained approval to conduct the survey from an ethics review board or a country-specific equivalent regulatory body.

This study included adolescents aged 11, 13 and 15 years (13.59 ± 1.64 years) from 34 countries which had data on VPA, a family affluence scale (FAS), age and gender for all three survey cycles (Austria, Belgium/Flemish, Canada, Croatia, the Czech Republic, Denmark, Estonia, Finland, France, Germany, Greece, Greenland, Hungary, Iceland, Ireland, Israel, Italy, Latvia, Luxembourg, the Netherlands, Norway, Poland, Romania, Russia, Slovakia, Slovenia, Spain, Sweden, Switzerland, Ukraine, Macedonia, England, Scotland and Wales). Samples were nationally representative and included a total of 501,647 children (in 2006: *n* = 167,176; 51.6% girls, in 2010: *n* = 172,707; 51.5% girls; in 2014: *n* = 161,764; 51.6% girls).

### Survey items

#### Vigorous physical activity (VPA)

VPA was assessed by the two following items.

- Outside school hours: How often do you usually exercise in your free time so much that you get out of breath or sweat? There were seven response categories: every day, four–six times a week, two-three times a week, once a week, once a month, less than once a month and never.
- Outside school hours: How many hours a week do you usually exercise in your free time so much that you get out of breath or sweat? The possible response categories were: none, about half an hour, about one hour, about two-three hours, about four–six hours, about seven hours or more.

The two items were combined into a single variable for VPA that reflects the VPA recommendations for children and adolescents (Tremblay et al. 2011; World Health Organization 2010). Therefore, the cut-off point was set to at least four–six times a week and at least about half an hour of VPA per week (Samdal et al. 2007). The reliability and validity of these items were considered to be acceptable among adolescents (Booth et al. 2001).

#### Family affluence scale (FAS)

Family affluence was measured using FAS. FAS was developed to measure material affluence as an indicator of family socio-economic status (Hartley et al. 2016). In the survey years 2006 and 2010, the FAS indicator included four items, but because of changes in living conditions and technology in recent decades and limitations concerning the categorization into low or high FAS, a new six-item FAS approach was developed, validated and used in the 2014 survey (Currie et al. 2008, 2014; Hartley et al. 2016; Torsheim et al. 2016).

FAS items in 2006 and 2010:

- Does your family own a car, van or truck? (No = 0; Yes, one = 1; Yes, two or more = 2);
- Do you have your own bedroom to yourself? (No = 0; Yes = 1);
- During the past 12 months, how many times did you go away on holiday with your family? (Not at all = 0; Once = 1; Twice = 2; More than twice = 3);
- How many computers does your family own? (None = 0; One = 1; Two = 2; More than two = 3).

FAS items in 2014:

- Does your family own a car, van or truck? (No = 0; Yes, one = 1; Yes, two or more = 2).
- Do you have your own bedroom to yourself? (No = 0; Yes = 1).
- How many computers does your family own (including laptops and tablets, not including game consoles and smartphones)? (None = 0; One = 1; Two = 2; More than two = 3).
- How many bathrooms (room with a bath/shower or both) are there in your home? (None = 0; One = 1; Two = 2; More than two = 3).
- Does your family have a dishwasher at home? (No = 0; Yes = 1).
- How many times did you and your family go abroad for a holiday/vacation last year? (Never = 0; Once = 1; Twice = 2; Three or more times = 3).

The FAS summary score (with higher scores indicating greater affluence) was converted to a continuous score, from 0 to 1 (Elgar et al. 2015) for each country and survey cycle separately. This score was split into three categories for each country and survey cycle separately as follows: 0–0.2000 = 1 (Low FAS), 0.2001–0.8000 = 2 (Medium FAS), 0.8001–1 = 3 (High FAS) (Inchley et al. 2016).

### Statistics

The analyses were conducted using IBM SPSS version 22. To describe the sample, we used logistic regression analyses (enter method) comparing gender, age groups, FAS categories and survey cycles in terms of meeting the VPA recommendations. The Pearson *χ*^2^ test was used to evaluate the changes between the selected years in the proportion of children who achieved the VPA recommendations by age group, gender and FAS categories.

The significance of trends over time was tested for each individual country and also for all countries combined using logistic regression analysis, the enter method (dependent variable: met VPA recommendations (yes/no, independent categorical variable (year of survey 2006, 2010, 2014) and controlled for age group (11, 13, and 15 years). The analyses were stratified by gender and FAS categories. For all analyses, the level of significance was set to 0.05.

## Results

Overall, girls were less likely to meet the VPA recommendations when compared to boys. An inverse association between age and meeting the VPA recommendations was found. In contrast, a positive association between FAS and VPA was observed; in that, adolescents from more affluent families were more likely to meet the VPA recommendations (Table 1). For the combined sample across all countries, a small but significant increase in VPA was observed between 2006 and 2014.

**Table 1:** Meeting vigorous physical activity recommendations within the overall sample and combined for all survey cycles and countries: Health Behaviour in School-aged Children study 2006–2014 in 34 countries in Europe and North America

Variable				
	Categories	%	OR	95% CI
Gender	Boys	48.5	Ref.	
	Girls	32.5	0.511***	0.505–0.517
Age category	11	45.8	Ref.	
	13	40.7	0.803***	0.792–0.815
	15	34.7	0.618***	0.609–0.627
FAS	Low	34.2	Ref.	
	Medium	39.7	1.241***	1.223–1.261
	High	47.9	1.708***	1.677–1.740
Survey years	2006	39.7	Ref.	
	2010	39.5	0.977**	0.963–0.991
	2014	41.8	1.095***	1.079–1.110

Legend: *FAS* Family Affluence Scale, *OR* odds ratio, *CI* confidence interval, % proportion of children who meet the vigorous physical activity recommendations in the selected category; *Ref*. reference group; **p < 0.01; ***p < 0.001

Table 2 shows trends in VPA in terms of FAS categories for all countries combined by age and gender. Between 2006 and 2014, we observe for the whole sample a statistically significant increase in meeting the VPA recommendations among girls from 31.3 to 34.8%, but not among boys, where we found no significant change. The most significant changes were in older girls from the medium- and high-FAS groups, where the proportion who reported meeting the VPA recommendations increased by more than six percentage points. In summary, for all FAS and age categories in girls except low-FAS 11-year-olds, an increase in VPA was reported between 2006 and 2014 (see Table 2). Among low-FAS boys, there was a significant decrease (or no change) in VPA between 2006 and 2014, whereas VPA increased among boys in the medium- and high-FAS groups over the same time period. These increases among boys were smaller compared to those among girls.

**Table 2:** Trends in meeting vigorous physical activity recommendations for all countries combined, by age and gender: Health Behaviour in School-aged Children study 2006–2014 in 34 countries in Europe and North America

Variable	Age	Boys						Girls					
		Year of survey			Trend			Year of survey			Trend		
		2006	2010	2014	2006/2010	2010/2014	2006/2014	2006	2010	2014	2006/2010	2010/2014	2006/2014
	Years	%	%	%	*p*	*p*	*p*	%	%	%	*p*	*p*	*p*
Low FAS	11	48.3	47.0	46.3	0.193	0.490	**0.044**	34.2	34.2	34.3	0.947	0.892	0.944
	13	44.9	42.4	42.9	**0.016**	0.644	**0.046**	26.4	25.3	29.0	0.147	**< 0.001**	**0.001**
	15	39.3	39.1	40.6	0.860	0.128	0.177	19.9	18.9	22.6	0.120	**< 0.001**	**< 0.001**
Medium FAS	11	51.9	51.4	51.9	0.410	0.422	0.998	38.8	38.3	40.2	0.294	**< 0.001**	**0.009**
	13	50.1	47.1	49.1	**< 0.001**	**< 0.001**	0.081	31.2	30.0	35.0	**0.015**	**< 0.001**	**0.001**
	15	43.0	42.6	44.6	0.479	**< 0.001**	**0.003**	23.4	24.1	29.1	0.161	**< 0.001**	**< 0.001**
High FAS	11	58.6	58.5	60.4	0.911	**0.036**	0.051	46.5	46.0	49.2	0.593	**0.001**	**0.008**
	13	55.3	55.4	57.9	0.865	**0.005**	**0.004**	37.8	38.7	44.0	0.363	**< 0.001**	**< 0.001**
	15	48.2	50.2	51.3	**0.042**	0.211	**0.002**	30.2	32.4	36.6	**0.017**	**< 0.001**	**< 0.001**
All		48.7	47.9	49.0	**0.001**	**< 0.001**	0.124	31.3	31.4	34.8	0.537	**< 0.001**	**< 0.001**

Legend: *FAS* Family Affluence Scale, *p* statistical significance based on Pearson *χ*^2^; % proportion of children who met the recommendations for vigorous physical activity

Significant results are shown in bold

### Time trends in meeting VPA recommendations by FAS across countries for boys

Among low-FAS boys, a significant increase in VPA was observed between 2006 and 2014 in Austria, Romania, Slovenia and Spain (Table 3). Over the same time period, a significant decrease in VPA was found in four countries (England, Italy, Slovakia and Wales).

**Table 3:**
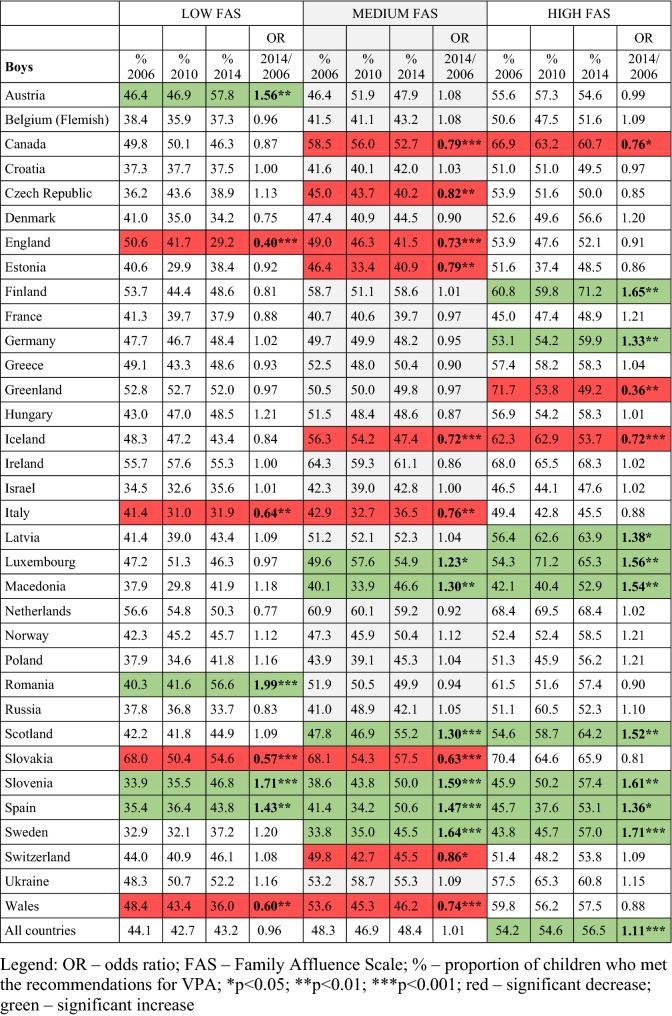
Odds ratios and prevalence (%) for meeting vigorous physical activity recommendations by family affluence scale group and country in boys (adjusted for age category): Health Behaviour in School-aged Children study 2006–2014 in 34 countries in Europe and North America (Color table online)

There was high variability between countries in the proportion of low-FAS boys meeting the VPA recommendations, with the lowest proportion observed in England in 2014 (29.2%) and the highest proportion in Slovakia in 2006 (68.0%) (Table 3). In the majority of countries, VPA was lower among low-FAS boys compared with boys in the medium- and high-FAS categories.

Among medium-FAS boys, a significant increase in VPA was observed in six countries (Luxembourg, Macedonia, Scotland, Slovenia, Spain and Sweden), whereas a decreasing trend in VPA was observed in nine countries (Canada, the Czech Republic, England, Estonia, Iceland, Italy, Slovakia, Switzerland and Wales). The lowest proportion of medium-FAS boys who met the VPA recommendations was observed in Italy in 2010 (32.7%), and the highest proportion was in Slovakia in 2006 (68.1%) (Table 3).

In the high-FAS category, a positive trend in VPA was observed in nine countries (Finland, Germany, Latvia, Luxembourg, Macedonia, Slovenia, Spain, Sweden and Scotland) and a significant decrease in three countries (Canada, Greenland and Iceland). The prevalence ranged from 37.4% of high-FAS boys in Estonia in 2010 to 71.7% in Greenland in 2006.

Across all countries combined, there was a significant positive increase in VPA in high-FAS boys but no change among low- or medium-FAS boys. Additionally, 13 countries showed no significant change in VPA in any FAS group.

### Time trends in meeting VPA guidelines across countries for girls

For low-FAS girls, a significant increase was reported between 2006 and 2014 in ten countries (Austria, the Czech Republic, Finland, Hungary, Macedonia, Norway, Romania, Slovenia, Spain and Sweden) and a significant decrease was observed in VPA in Slovakia, England and Wales (Table 4). Overall, fewer low-FAS girls meet the VPA recommendations than girls in the medium- and high-FAS categories. The prevalence among low-FAS girls was lowest in Macedonia in 2010 (8.5%) and highest in Finland in 2014 (51.8%).

**Table 4:**
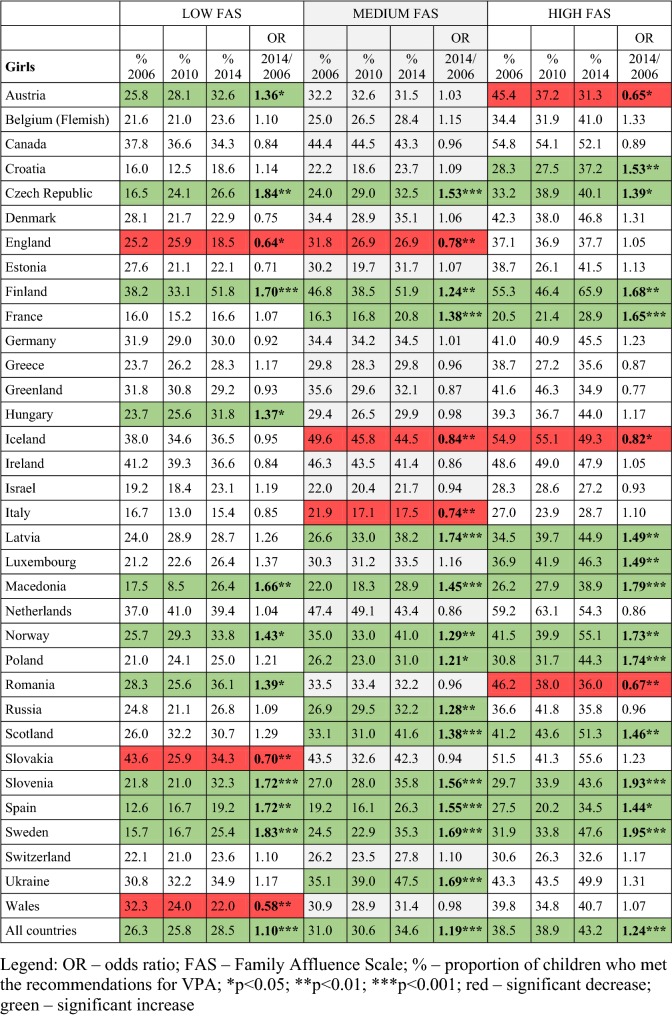
Odds ratios and prevalence (%) for meeting the vigorous physical activity recommendations, by family affluence scale and country or region in girls (adjusted for age category): Health Behaviour in School-aged Children study 2006–2014 in 34 countries in Europe and North America (Color table online)

In girls from the medium-FAS category, a significant increase was observed in 13 countries (the Czech Republic, Finland, France, Latvia, Norway, Macedonia, Poland, Russia, Scotland, Slovenia, Spain, Sweden and Ukraine), and only three countries (England, Iceland and Italy) showed a decreasing trend between 2006 and 2014. Among medium-FAS girls, the prevalence was lowest in Spain in 2010 (16.1%) and highest in Finland in 2014 (51.9%).

Among high-FAS girls, there were positive trends in 13 countries (Croatia, the Czech Republic, Finland, France, Latvia, Luxembourg, Macedonia, Norway, Poland, Scotland, Slovenia, Spain and Sweden) and a significant decrease in Austria, Iceland and Romania. The lowest proportion of girls who met the VPA recommendations was in Spain in 2010 (20.2%) and the highest proportion in Finland in 2014 (65.9%).

For all countries combined, girls in all FAS categories showed a small but significant increase in meeting the VPA recommendations, but this increase was greatest among high-FAS girls. Furthermore, seven countries showed increases across all FAS groups and 11 countries showed no significant change in VPA in any FAS group (see Table 4).

## Discussion

This study presents cross-national trends in VPA and shows how social inequalities in participation in VPA have changed over time. From 2006 to 2014, the proportion of adolescents meeting the VPA recommendations rose slightly, but these trends varied according to gender and socio-economic groups among the 34 participating countries. Across the 2006, 2010 and 2014 surveys, the observed increase was stronger in girls. However, the prevalence of VPA for girls was generally lower than for boys, even in 2014. From a socio-economic perspective, our study showed that medium- and high-FAS adolescents in most countries are more involved in VPA than lower-FAS groups. Furthermore, we observed an increase in meeting the VPA recommendations between 2006 and 2014 for boys and girls from higher FAS categories more often than low-FAS adolescents.

Previous reviews of trends in PA among children and adolescents have concluded that there is a little evidence of a decline in overall PA in recent decades (Booth et al. 2015; Ekelund et al. 2011). As overall PA is challenging to monitor, it is suggested that specific domains and types of PA should be studied separately. Our measure is related to VPA outside school hours, hence more related to leisure time, extracurricular activities and sports, as well as vigorous intensity. By comparing our data with earlier studies, we found similar patterning by age and gender, with studies having consistently reported that boys are more active than girls and that PA declines with age (Finne et al. 2011; Kalman et al. 2015; Sigmundová et al. 2014; Trost et al. 2002).

Socio-economically, we found that medium- and high-FAS adolescents in most countries are more involved in VPA than lower-FAS ones and similar findings have been reported previously (Brodersen et al. 2007; Chzhen et al. 2018). Revealing a significant increase between 2006 and 2014 in meeting, the VPA recommendations for girls from all socio-economic categories can be considered to be a positive finding, given the fact that MVPA generally stagnates (Inchley et al. 2017). However, the prevalence of meeting the VPA recommendations, as well as the MVPA recommendation (at least 60 min daily World Health Organization 2010), is still lower for girls than for boys (Kalman et al. 2015) and the data show that many young people are not meeting the recommended levels.

Serious health-related problems can develop rapidly during adolescence and it has been shown in our study and others that adolescents from medium- and high-affluence families in most countries and regions have followed the VPA and MVPA recommendations more systematically than adolescents from low-affluence families (Inchley et al. 2016, 2017; Sigmund et al. 2018). Thus, adolescents from low-affluence families are most likely to be susceptible to the negative health outcomes associated with low levels of PA.

The results from the present study also provide important information for public health policy and practice. While the international recommendation highlights the importance of MVPA (World Health Organization 2010) evidence suggests that higher-intensity PA may be particularly beneficial for adolescents because it is more strongly associated with cardio-respiratory fitness and health outcomes compared with MVPA (Hay et al. 2012; Marques et al. 2015; Parikh and Stratton 2011; Steele et al. 2009). Thus, future PA recommendations for young people may focus more explicitly on an increase in the time spent on VPA.

In order to increase the prevalence of VPA, we recommend promoting involvement in organized sports for both girls and boys. Previous studies found that adolescents involved in organized sport spend significantly more time on VPA and MVPA than their non-involved counterparts (Machado-Rodrigues et al. 2012; Marques et al. 2016). Therefore, participation in organized sports can provide an important opportunity for increasing VPA.

From the policy perspective (Bull et al. 2014; Milton and Bauman 2015; Sember et al. 2016; Tremblay et al. 2016), the results also imply some indications for a potential association between the level of participation in VPA and national policy-promoting PA and sport. Slovenia, for example, where significant progress in the level of VPA was reported in all FAS and gender categories, seems to be a good example of a country successfully implementing public policy-promoting PA in different sectors such as health, education, sport or transport (Bull et al. 2014). The Slovenian National Programme of Sport 2014–2023 or the National Program of Nutrition and Physical Activity for Health 2015–2025 are being implemented using an evidence-based policy planning approach, and are in line with previous Slovenian national strategies and thus have been consistent over the past two decades (Sember et al. 2016). A similar picture could be seen in some gender and FAS categories in the Nordic countries, such as Norway or Finland, where PA is of high importance on the political agenda. Those countries also indicated some promising results in policy implementation and evaluation (Bull et al. 2014; Tremblay et al. 2016).

### Strengths and limitations

A large sample size and a cross-national approach can be considered as the major strengths of this study. The HBSC study is based on a standardized methodological protocol using the same items in all participating countries. In addition, the reported VPA trends over 34 countries reflect a broad range of economic, geographic, political and socio-cultural contexts. However, the conclusions drawn from the trend analyses presented here need to be considered in the light of limitations. First, the HBSC study used self-reported measures of VPA and FAS items, which might lead to potential bias or misclassification, although previous studies have reported acceptable reliability and validity for these measures (Booth et al. 2001; Currie et al. 2008, 2014; Hartley et al. 2016; Hobza et al. 2017; Torsheim et al. 2016). Second, social desirability can potentially affect self-reported data. However, the magnitude, direction and trend-related variations in these potential effects remain unknown. Adolescents participating in the HBSC study are assured of anonymity and confidentiality, which could have helped minimize the effect of social desirability in the participants’ responses.

To conclude, despite the highlighted limits of the study, we demonstrated differences in the VPA habits of adolescents across Europe, Israel and North America. Marked social inequalities exist, with adolescents from medium- and high-affluence families being more likely to take part in VPA than those from low-affluence families. These fundamental inequalities occur at a critical developmental stage of the life course and are likely to have substantial implications for the future health and well-being of adolescents growing up in more disadvantaged circumstances. Barriers to participation in VPA must be identified in order to make opportunities more accessible to those young people who could benefit most. The increases in VPA among girls in some countries are particularly encouraging and may reflect targeted approaches and successful efforts in public policy which have been applied at the national level. Further research is required to identify effective interventions which successfully reach girls on a larger scale, given that they are more likely than boys to disengage from sport and PA during their adolescent years. There is, however, a low rate of VPA among both boys and girls in many countries, which shows the potential for engaging in the promotion of VPA. This lack of VPA may also contribute to the rise in child obesity and general lower health, especially among low-affluence adolescents.
